# Reference Values for Cardiorespiratory Fitness and Incidence of Type 2 Diabetes

**DOI:** 10.2188/jea.JE20130076

**Published:** 2014-01-05

**Authors:** Ryoko Kawakami, Susumu S. Sawada, Munehiro Matsushita, Takashi Okamoto, Koji Tsukamoto, Mitsuru Higuchi, Motohiko Miyachi

**Affiliations:** 1 Graduate School of Sport Sciences, Waseda University, Tokorozawa, Saitama, Japan; 1 早稲田大学大学院 スポーツ科学研究科; 2 Department of Health Promotion and Exercise, National Institute of Health and Nutrition, Tokyo, Japan; 2 独立行政法人国立健康・栄養研究所 健康増進研究部; 3 Department of Safety and Health, Tokyo Gas Co. Ltd., Tokyo, Japan; 3 東京ガス株式会社 安全健康・福利室; 4 Faculty of Sport Sciences, Waseda University, Tokorozawa, Saitama, Japan; 4 早稲田大学 スポーツ科学学術院

**Keywords:** hyperglycemia, maximal oxygen uptake, risk factor, cohort study, Japanese

## Abstract

**Background:**

In “Physical Activity Reference for Health Promotion 2013” the Japan Ministry of Health, Labour and Welfare publication gives reference values for cardiorespiratory fitness (CRF) required for good health. We examined the associations between the CRF reference values and incidence of type 2 diabetes.

**Methods:**

This prospective cohort study enrolled 4633 nondiabetic Japanese men aged 20 to 39 years at baseline. CRF was measured using the cycle ergometer test, and maximal oxygen uptake was estimated. On the basis of the CRF reference value, participants were classified into 2 groups: those with values less than the reference value (under-RV) and those with values equal to or greater than reference value (over-RV). Hazard ratios (HRs) and 95% CIs for incident type 2 diabetes were estimated using a Cox proportional hazards model.

**Results:**

A total of 266 participants developed type 2 diabetes during the 14 years of follow-up. As compared with the under-RV group, the over-RV group had a significantly lower multivariable-adjusted HR for type 2 diabetes (HR 0.67; 95% CI, 0.51–0.89). In receiver operating characteristic analysis, the optimal CRF cut-off value for predicting incident type 2 diabetes was 10.8 metabolic equivalents (sensitivity, 0.64; specificity, 0.64), which was close to the CRF reference value of 11.0 metabolic equivalents.

**Conclusions:**

The reference CRF value appears to be reasonably valid for prevention of type 2 diabetes, especially among Japanese men younger than 40 years. Development of type 2 diabetes can be prevented by maintaining a CRF level above the reference value.

## Abstract (ja)

**【背景】:**

日本では厚生労働省より「健康づくりのための身体活動基準2013」が策定され、健康づくりのために必要な全身持久力の基準値が示されている。本研究は、この全身持久力の基準値と2型糖尿病罹患との関連について縦断的に検討することを目的とした。

**【方法】:**

対象者は、糖尿病でない20～39歳の日本人男性4633名であった。自転車エルゴメータを用いた最大下運動負荷試験によって最大酸素摂取量が推定され、全身持久力の基準値をもとに達成者と未達成者の2群に分けられた。Cox比例ハザードモデルを用いて2型糖尿病罹患のハザード比および95%信頼区間を求めた。

**【結果】:**

14年間の追跡期間中、266名が2型糖尿病に罹患した。全身持久力の基準値達成者は、未達成者と比較して、多変量調整ハザード比が統計上有意に低かった（ハザード比0.67、95%信頼区間0.51-0.89）。Receiver Operating Characteristic曲線による解析の結果、2型糖尿病発症の予測に向けた最大酸素摂取量のカットオフ値は10.8メッツ（感度0.64、特異度0.64）となり、40歳未満の男性の全身持久力の基準値11.0メッツと近似値であった。

**【結論】:**

全身持久力の基準値は、特に40歳未満の日本人男性において、2型糖尿病予防に対しておおよそ妥当な値であると考えられた。全身持久力の基準値以上の値を保持することによって、2型糖尿病の発症が抑制できる可能性が示唆された。

## INTRODUCTION

The International Diabetes Federation reported that 366 million people worldwide and 10.67 million Japanese had diabetes in 2011.^1^ With the rapidly increasing prevalence of this condition globally, the number is predicted to rise to 552 million by 2030.^2^

Prospective cohort studies reported a lower risk of incident type 2 diabetes among individuals with high levels of cardiorespiratory fitness (CRF).^3^^–^^12^ Therefore, maintaining higher CRF is an important factor in preventing type 2 diabetes. However, to our knowledge, no epidemiologic studies have quantitatively examined the CRF level needed to prevent future type 2 diabetes.

In March 2013 the Japan Ministry of Health, Labour and Welfare published the “Physical Activity Reference for Health Promotion 2013,” which recommends sex-specific and age-group–specific reference CRF values required for good health. The values are based on the results of systematic reviews and meta-analyses of the associations of CRF with noncommunicable diseases (including type 2 diabetes and motor disorders) and death.^13^ However, the associations between the reference values and incidence of type 2 diabetes have not yet been studied, and the reference values may need to be validated. In addition to physical fitness, there are several risk factors potentially associated with type 2 diabetes, including age,^14^ obesity,^15^ smoking,^16^ drinking,^17^ and family history.^18^ A limited number of epidemiologic studies have examined the interactions of these risk factors with CRF, indicating that it is important to evaluate associations between CRF reference values and incidence of type 2 diabetes among individuals with these risk factors.

We used a longitudinal design to examine the associations between reference CRF values recommended in “Physical Activity Reference for Health Promotion 2013” and incidence of type 2 diabetes among Japanese male workers. In addition, we investigated associations of reference CRF values with multiple risk factors for type 2 diabetes.

## METHODS

### Participants

The participants were employees of Tokyo Gas Company, which supplies natural gas to the Tokyo area. All employees received annual health checkups and completed a health-related questionnaire in accordance with the Industrial Safety and Health Law.

The participants for this study were 5984 male employees aged 20 to 40 years who had participated in the health checkup of 1985. Among these 5984 men, 335 were excluded because they were found at the health checkup to have at least 1 of the following conditions: diabetes (*n* = 102), cardiovascular disease including hypertension (*n* = 228), tuberculosis (*n* = 3), and gastrointestinal disease (*n* = 9). We also excluded 904 men who did not perform or complete a submaximal exercise test, due to orthopedic disease, poor general health, absence from work, or other reasons. In addition, 112 men aged 40 years at baseline were excluded because the presently investigated reference value pertains only to men aged 18 to 39 years. Ultimately, 4633 men were included in the present analysis and were followed until June 1999 for development of type 2 diabetes.

This study was approved by the Ethical Review Board of the National Institute of Health and Nutrition.

### Clinical examination

The annual health checkup, including measurement of height, body weight, and blood pressure, has been conducted since 1985. Body mass index (BMI) was calculated as weight divided by the height squared (kg/m^2^). Blood pressure was measured by the auscultatory method with a mercury sphygmomanometer; diastolic pressure was recorded when no sound could be heard.

Cigarette smoking, alcohol intake, and family history of diabetes were assessed using a questionnaire administered before the start of the submaximal exercise test.

### Cardiorespiratory fitness test

The participants underwent a submaximal exercise test on a cycle ergometer to assess CRF. The exercise test consisted of 2 to 3 progressively increasing 4-minute exercise stages. The initial exercise loads were 98 and 86 watts for participants aged 20 to 29 and 30 to 39 years, respectively. Pedaling rate was kept constant at 50 rpm. Heart rate was calculated from the RR interval on an electrocardiogram, and 85% of the age-predicted maximal heart rate (220 − age [years]) was set as the target heart rate. Among all age groups, exercise load was increased by 37 watts for each stage, until heart rates during the course of the exercise reached the target heart rate or until completion of the third stage. On the basis of the exercise rate during the last 1 minute of the final stage completed and the heart rate obtained from the last 10 seconds of exercise, maximal oxygen uptake (VO_2_max) was estimated using the Åstrand-Ryhming nomogram^19^ and Åstrand age-correction factors.^20^

The participants were classified into 2 groups based on the CRF reference value in “Physical Activity Reference for Health Promotion 2013”^13^: those with values less than the reference value (under-RV) group and those with values equal to or greater than the reference value (over-RV) group (Table 1).

**Table 1.** Reference values for cardiorespiratory fitness for men

	18–39 years	40–59 years	60–69 years
VO_2_max (METs)	11.0	10.0	9.0

Abbreviations: MET, metabolic equivalent; VO_2_max, maximal oxygen uptake.

### Diagnosis of type 2 diabetes

During 1985–1999, participants were followed for development of type 2 diabetes, which was diagnosed if any of the following 3 diagnostic parameters was noted: (1) a plasma glucose level greater than 200 mg/dl (11.1 mmol/l) at 2 hours on an oral glucose tolerance test (conducted among men with urinary glucose detected at a follow-up annual health checkup), (2) self-reported current therapy with hypoglycemic medication (insulin or oral hypoglycemic agent), when interviewed at the subsequent health checkup, or (3) a fasting plasma glucose greater than 126 mg/dl (7.0 mmol/l). The criterion for fasting plasma glucose level in the diagnosis of type 2 diabetes was based on the diagnostic guidelines of the American Diabetes Association^21^ and Japan Diabetes Society.^22^ However, the fasting plasma glucose test has been used only since 1988 among participants aged 35 years and those aged 40 years or older.

### Statistical analysis

We compared baseline characteristics of participants according to CRF category (under-RV group vs over-RV group) using the unpaired *t*-test for continuous variables and Kruskal-Wallis test for categorical variables, as appropriate. We used Cox proportional hazards models to estimate hazard ratios (HRs) and 95% CIs for incident type 2 diabetes, adjusted for age (continuous variable), BMI (continuous variable), systolic blood pressure (continuous variable), family history of diabetes (present or not), cigarette smoking (nonsmokers, 1–20 cigarettes/day; smokers, ≥21 cigarettes/day), and alcohol intake (nondrinkers, 1–45 g/day; drinkers, ≥46 g/day) in a multivariable model according to CRF category. Family history of diabetes was defined as the known presence of family members with diabetes in any of 3 generations, as determined by self-report on the health questionnaire. Receiver operating characteristic (ROC) analysis was used to identify the optimal cut-off value for VO_2_max in predicting type 2 diabetes. The optimal cut-off value was calculated by determining the shortest distance between the ROC curve and the upper left corner of the graph. A 2-tailed *P* value less than 0.05 was considered statistically significant. All statistical analyses were performed with SPSS Statistics version 20.0 for Windows (IBM Japan, Tokyo, Japan).

## RESULTS

The mean age of the participants was 31 years (range, 20–39 years) at baseline. During a median of 14.1 years of follow-up (62 937 person-years), 266 men developed type 2 diabetes. During the follow-up period, a total of 169 participants were censored due to death or retirement.

Table 2 shows the baseline characteristics of the participants according to CRF category. Mean VO_2_max was 11.7 metabolic equivalents (METs) (median 11.4 METs). Men in the over-RV group were younger and had lower weight, BMI, systolic blood pressure, and diastolic blood pressure than did those in the under-RV group. The over-RV group also had lower rates of smoking, drinking, and family history of diabetes.

**Table 2.** Baseline characteristics according to cardiorespiratory fitness category

	Under-RV	Over-RV	All	*P* value
*n*	1946	2687	4633	
VO_2_max (METs)	9.7 (0.9)	13.2 (1.8)	11.7 (2.3)	<0.001
Age (years)	33 (4)	30 (5)	31 (5)	<0.001
Height (cm)	169.8 (5.4)	169.3 (5.5)	169.5 (5.5)	0.002
Weight (kg)	68.8 (8.4)	63.6 (7.4)	65.8 (8.2)	<0.001
Body mass index (kg/m^2^)	23.9 (2.6)	22.2 (2.2)	22.9 (2.5)	<0.001
Systolic blood pressure (mm Hg)	128.1 (11.4)	123.5 (11.4)	125.4 (11.6)	<0.001
Diastolic blood pressure (mm Hg)	75.2 (8.8)	70.8 (8.7)	72.6 (9.0)	<0.001
Smokers (%)	69.8	65.7	67.4	0.003
Drinkers (%)	71.4	67.3	69.0	0.003
Family history of diabetes (%)	24.0	20.1	21.7	0.001

Abbreviations: MET, metabolic equivalent; RV, reference value.

Data are expressed as mean (SD) for variables or as percentages of participants.

**Figure 1.**
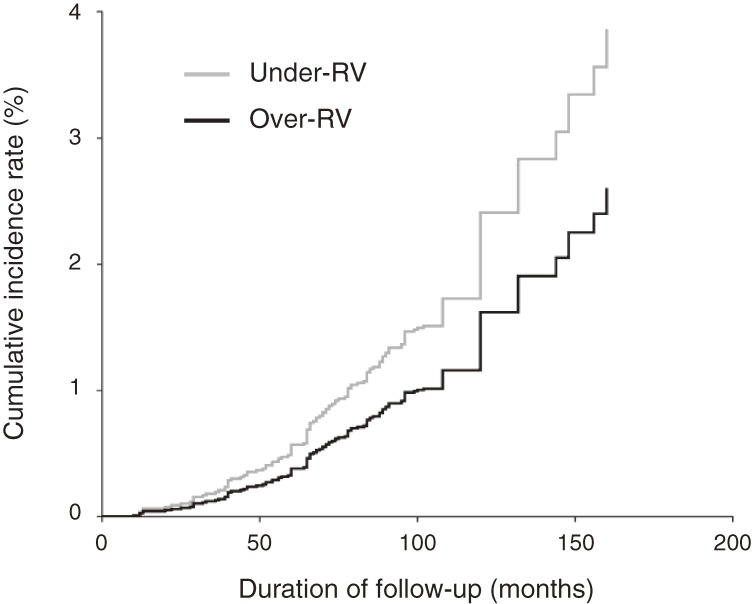
Multivariable-adjusted cumulative incidence curve for type 2 diabetes according to cardiorespiratory fitness category (adjusted for age, body mass index, systolic blood pressure, cigarette smoking, alcohol intake, and family history of diabetes). RV, reference value.

The HRs and 95% CIs for incidence of type 2 diabetes, by CRF category, are shown in Table 3. After adjustment for age, systolic blood pressure, cigarette smoking, alcohol intake, and family history of diabetes, the HR (model 1) for the over-RV group was 0.44 (95% CI, 0.34–0.58), when compared with the under-RV group. After further adjustment for BMI (model 2), the HR for type 2 diabetes remained significantly lower for the over-RV group (0.67; 95% CI, 0.51–0.89). Figure 1 shows the multivariable-adjusted cumulative incidence curve for type 2 diabetes according to CRF category.

**Table 3.** Hazard ratios (HRs) for incident type 2 diabetes according to cardiorespiratory fitness category

Category	*n*	Person-years of follow-up	No. of cases	Age-adjusted HR (95% CI)	Model 1^a^ HR (95% CI)	Model 2^b^ HR (95% CI)
Under-RV	1946	26 091	183	1.00 (Reference)	1.00 (Reference)	1.00 (Reference)
Over-RV	2687	36 846	83	0.36 (0.28–0.48)	0.44 (0.34–0.58)	0.67 (0.51–0.89)
*P* value				<0.001	<0.001	0.005

Abbreviation: RV, reference value.

^a^Model 1: Adjusted for age, systolic blood pressure, cigarette smoking, alcohol intake, and family history of diabetes.

^b^Model 2: Adjusted for Model 1 covariates plus body mass index.

**Figure 2.**
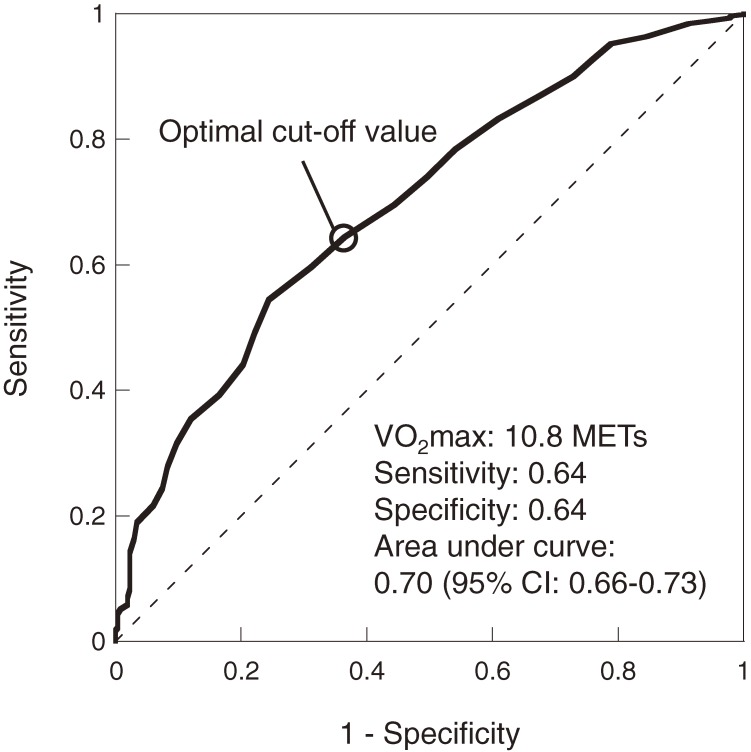
Receiver operating characteristic curve for incidence of type 2 diabetes against baseline maximal oxygen uptake.

Figure 2 illustrates the results of ROC analysis of the incidence of type 2 diabetes, against baseline VO_2_max levels. The area under the ROC curve was 0.70 (95% CI, 0.66–0.73), and the optimal cut-off value was 10.8 METs (sensitivity, 0.64; specificity, 0.64).

We then investigated HRs and 95% CIs for incident type 2 diabetes according to CRF category in analysis stratified by age (20–29 or 30–39 years), BMI (<25 or ≥25), cigarette smoking (nonsmokers or smokers), alcohol intake (nondrinkers or drinkers), and family history of diabetes (yes or no) (Table 4). The risk for type 2 diabetes among the over-RV group was significantly lower than in the under-RV group, irrespective of age, BMI, or alcohol intake. Although there was no significant difference in relation to CRF among smokers and men with a family history of diabetes, the results suggest that risk was lower among the over-RV group than among the under-RV group.

**Table 4.** Hazard ratios (HRs) for incident type 2 diabetes according to cardiorespiratory fitness category in analysis stratified by potential risk factors

	*n*	Person-years of follow-up	No. of cases	Multivariable HR (95% CI)^a^	*P* value		*n*	Person-years of follow-up	No. of cases	Multivariable HR (95% CI)^a^	*P* value
Age 20–29 years						Age 30–39 years					
Under-RV	474	6374	37	1.00 (Reference)		Under-RV	1472	19 717	146	1.00 (Reference)	
Over-RV	1114	15 375	17	0.37 (0.20–0.71)	0.003	Over-RV	1573	21 471	66	0.73 (0.54–0.99)	0.045

Body mass index < 25						Body mass index ≥ 25					
Under-RV	1354	18 469	76	1.00 (Reference)		Under-RV	592	7623	107	1.00 (Reference)	
Over-RV	2407	33 108	58	0.55 (0.39–0.79)	0.001	Over-RV	280	3738	25	0.57 (0.37–0.89)	0.013

Nonsmokers						Smokers					
Under-RV	587	7927	53	1.00 (Reference)		Under-RV	1359	18 165	130	1.00 (Reference)	
Over-RV	922	12 705	23	0.56 (0.33–0.95)	0.030	Over-RV	1765	24 141	60	0.73 (0.52–1.01)	0.056

Nondrinkers						Drinkers					
Under-RV	556	7508	36	1.00 (Reference)		Under-RV	1390	18 583	147	1.00 (Reference)	
Over-RV	879	12 079	13	0.45 (0.23–0.89)	0.022	Over-RV	1808	24 767	70	0.73 (0.53–0.99)	0.041

No family history of diabetes						Family history of diabetes					
Under-RV	1479	20 072	89	1.00 (Reference)		Under-RV	467	6019	94	1.00 (Reference)	
Over-RV	2148	29 543	37	0.61 (0.40–0.92)	0.017	Over-RV	539	7303	46	0.71 (0.49–1.05)	0.087

Abbreviation: RV, reference value.

^a^Adjusted for all items in table.

## DISCUSSION

In this longitudinal study of Japanese men, we investigated the associations of the CRF reference value in the “Physical Activity Reference for Health Promotion 2013” with incident type 2 diabetes and its potential risk factors.

A total of 266 participants developed type 2 diabetes during the 14 years of follow-up. As compared with the under-RV group, the over-RV group had a significantly (33%) lower HR for type 2 diabetes after adjusting for age, blood pressure, cigarette smoking, alcohol intake, family history of diabetes, and BMI, which suggests that the risk of developing type 2 diabetes is reduced by maintaining a CRF level above the reference values recommended in the “Physical Activity Reference for Health Promotion 2013.”

The association between CRF and incident type 2 diabetes has been examined in several prospective cohort studies.^3^^–^^12^ Lynch et al^10^ reported an inverse relationship between CRF, as determined by expired gas analysis during a maximal bicycle ergometer test, and incident type 2 diabetes in middle-aged Finnish men. Wei et al^4^ revealed a significant dose-response relationship between CRF assessed using the modified Balke protocol and incident type 2 diabetes among men younger than 45 years and those aged 45 years or older. Additionally, Carnethon et al^8^ reported that young men and women (age 18–30 years) with low CRF had a significantly higher risk of developing type 2 diabetes. Consistent with these previous findings, we found that high CRF was associated with lower risk of incident type 2 diabetes among Japanese male employees younger than 40 years. This suggests that the incidence of type 2 diabetes can be reduced by achieving the CRF reference values recommended in “Physical Activity Reference for Health Promotion 2013.”

In our ROC analysis, the optimal CRF cut-off value for predicting incident type 2 diabetes was 10.8 METs (sensitivity, 0.64; specificity, 0.64), which is close to the reference CRF value of 11.0 METs (for men aged 18–39 years) recommended in “Physical Activity Reference for Health Promotion 2013.” This suggests that the guidelines’ reference CRF value for men younger than 40 years may be reasonably valid in the prevention of type 2 diabetes. When used as a cut-off value, the reference CRF value yielded a sensitivity of 0.60 and a specificity of 0.69, indicating that testing—independent of whether the reference CRF value is achieved—predicts incident type 2 diabetes with at least 60% accuracy. In a longitudinal study of men and women aged 18 to 69 years, Katzmarzyk et al^3^ reported a cut-off value of 11.3 METs (sensitivity, 60.6; specificity, 59.0) for predicting incident type 2 diabetes, based on estimated VO_2_max during a step test (modified Canadian Aerobic Fitness Test). The cut-off value computed in our study was similar to that previously reported value; however, because the present analysis was limited to men aged 20 to 39 years at baseline, additional epidemiologic evidence from more comprehensive, population-specific studies is needed.

In addition to physical fitness, there are several risk factors associated with type 2 diabetes, including age,^14^ obesity,^15^ smoking,^16^ drinking,^17^ and family history.^18^ We performed stratified analyses to examine the relationships between observed associations and these risk factors and found that the over-RV group had a significantly lower risk of developing type 2 diabetes than the under-RV group, regardless of age, BMI, or drinking status. In analyses stratified by smoking status, nonsmokers in the over-RV group had a significantly lower HR, whereas smokers in the over-RV group had a tendency toward lower risk of diabetes (*P* = 0.056). Analyses stratified by family history of diabetes revealed that individuals in the over-RV group that had such a history had a nonsignificantly (*P* = 0.087) lower HR for type 2 diabetes, in contrast to individuals in the over-RV group without such a history, for whom the HR was significant (HR: 0.71 and 0.61, respectively). In a prospective cohort study,^6^ a combined analysis of parental history of diabetes and CRF showed that individuals with high CRF, even including the children of 2 diabetic parents, had a lower risk of incident type 2 diabetes than did those with low CRF. Although the presence of a family history of diabetes is a strong, unmodifiable risk factor for the development of diabetes, present and previous findings suggest that the risk of developing type 2 diabetes can be reduced by maintaining high CRF.

This study has several limitations. First, the participants were relatively young (20–39 years) healthy male workers, so it is unclear whether the observed associations are present among men aged 40 years or older and women. However, several studies found an inverse relationship between CRF level and development of type 2 diabetes among women and older middle-aged men.^4^^,^^10^^,^^12^ We plan to examine the validity of the CRF reference value for these populations in a future study. Second, CRF was estimated in this study from heart rate during submaximal exercise using the Åstrand-Ryhming nomogram. However, Teräslinna et al^23^ reported a correlation coefficient of 0.92 between estimated (using the same procedures as ours) and directly measured CRF values. Thus, we assume that the estimated values used in this study as a measure of whole-body endurance capacity are sufficiently accurate. Finally, CRF was based on data obtained at the baseline examination, and possible changes in CRF during the follow-up period were not considered.

In conclusion, the reference CRF values appear to be reasonably valid for prevention of type 2 diabetes, especially among Japanese men younger than 40 years. Development of type 2 diabetes can be prevented by maintaining a CRF level above the reference values recommended in “Physical Activity Reference for Health Promotion 2013.”

## ONLINE ONLY MATERIALS

Abstract in Japanese.
