# Supplementary material for: Reference Values for Cardiorespiratory Fitness and Incidence of Type 2 Diabetes
Source: J Epidemiol. 2014 Jan 5;24(1):25–30. doi: 10.2188/jea.JE20130076 (PMC3872521; doi:10.2188/jea.JE20130076)
Supplement: Abstract in Japanese. [file je-24-025-s001.pdf]

全身持久力の基準値と 2 型糖尿病罹患

川上諒子 1,2、澤田亨 2、松下宗洋 1,2、岡本隆史 3、塚本浩二 3、樋口満 4、宮地元彦 2

1 早稲田大学大学院 スポーツ科学研究科、2 独立行政法人国立健康・栄養研究所 健康増進研究部、3 東京ガス株式会社 安全健康・福利室、4 早稲田大学 スポーツ科学学術院

【背景】日本では厚生労働省より「健康づくりのための身体活動基準 2013」が策定され、健康づくりのために必要な全身持久力の基準値が示されている。本研究は、この全身持久力の基準値と 2 型糖尿病罹患との関連について縦断的に検討することを目的とした。

【方法】対象者は、糖尿病でない 20～39 歳の日本人男性 4633 名であった。自転車エルゴメータを用いた最大下運動負荷試験によって最大酸素摂取量が推定され、全身持久力の基準値をもとに達成者と未達成者の 2 群に分けられた。Cox 比例ハザードモデルを用いて 2 型糖尿病罹患のハザード比および 95%信頼区間を求めた。

【結果】14 年間の追跡期間中、266 名が 2 型糖尿病に罹患した。全身持久力の基準値達成者は、未達成者と比較して、多変量調整ハザード比が統計上有意に低かった（ハザード比 0.67、95%信頼区間 0.51-0.89）。Receiver Operating Characteristic 曲線による解析の結果、2 型糖尿病発症の予測に向けた最大酸素摂取量のカットオフ値は 10.8 メッツ（感度 0.64、特異度 0.64）となり、40 歳未満の男性の全身持久力の基準値 11.0 メッツと近似値であった。

【結論】全身持久力の基準値は、特に 40 歳未満の日本人男性において、2 型糖尿病予防に対しておおよそ妥当な値であると考えられた。全身持久力の基準値以上の値を保持することによって、2 型糖尿病の発症が抑制できる可能性が示唆された。

キーワード：高血糖、最大酸素摂取量、危険因子、コホート研究、日本人
